# Mitigating Disparities in Prostate Cancer Survival Prediction Through Fairness‐Aware Machine Learning Models

**DOI:** 10.1002/cam4.71544

**Published:** 2026-01-27

**Authors:** Hyungrok Do, Rajesh Ranganath, Katie Murray, Madhur Nayan

**Affiliations:** ^1^ Department of Population Health New York University School of Medicine New York New York USA; ^2^ Center for Data Science New York University New York New York USA; ^3^ Courant Institute of Mathematical Sciences New York University New York New York USA; ^4^ Department of Urology, New York University School of Medicine New York New York USA; ^5^ Department of Urology, Bellevue Hospital New York City Health and Hospitals New York New York USA

**Keywords:** bias, fairness, machine learning, prostate cancer, survival

## Abstract

**Purpose:**

Prediction models can contribute to disparities in care by performing unequally across demographic groups. While fairness‐aware methods have been explored for binary outcomes, applications to survival analysis remain limited. This study compares two fairness‐aware deep learning survival models to mitigate racial disparities in predicting survival after radical prostatectomy for prostate cancer.

**Methods:**

We used the National Cancer Database to train deep Cox proportional hazards models for overall survival. Two fairness‐aware approaches, Fair Deep Cox Proportional Hazards Model (Fair DCPH) and Group Distributionally Robust Optimization Deep Cox Proportional Hazards Model (GroupDRO DCPH), were compared against a standard Deep Cox model (Baseline). Model fairness was assessed via cross‐group and within‐group concordance indices (C‐index).

**Results:**

Among 418,968 included patients, 78.5% were White, with smaller proportions of Black (13.2%), Hispanic (4.5%), Asian (1.9%), and Other (2.0%) patients. The baseline DCPH model achieved a cross‐group C‐index of 0.699 for White patients but showed reduced performance for Black (0.678) and Hispanic (0.689) patients. Fairness‐aware models improved cross‐group C‐indices; for Black patients, cross‐group C‐index increased to 0.692 (Fair DCPH) and 0.696 (GroupDRO DCPH); for Hispanic patients, to 0.693 and 0.697, respectively. Cross‐group C‐index also improved in the Asian subgroup, where the C‐index rose from 0.696 (Baseline DCPH) to 0.702 (Fair DCPH) and 0.707 (GroupDRO DCPH), with minimal performance loss observed for White patients.

**Conclusion:**

We benchmark two fairness‐aware survival models that address racial disparities in post‐prostatectomy survival prediction. These methods can be extended to other time‐to‐event models to ensure equitable care supported by fair prediction models.

## 1 Introduction

Prostate cancer is the most commonly diagnosed malignancy among males, with an estimated 313,780 new cases in the United States in 2025 [1]. Most patients present with localized disease, for which standard definitive treatment options include radical prostatectomy (RP) or radical radiotherapy, with or without hormonal therapy [2]. Among these, RP is more frequently utilized in the United States [3]. Predicting survival after RP is important for clinicians and patients because it can inform prognosis, guide subsequent therapy, and provide long‐term expectations.

Machine learning (ML) and artificial intelligence (AI)‐based approaches are increasingly being applied to develop predictive algorithms in healthcare due to their capability to identify complex, nonlinear patterns and optimize models with minimal human input [4, 5, 6]. However, growing evidence suggests that models developed using the approaches may demonstrate algorithmic unfairness, defined as a model whose decisions are skewed toward a particular group of people [7]. The consequences of an unfair model raise critical concerns in healthcare where biased predictions may contribute to diminished accuracy for certain populations [8, 9, 10, 11, 12] and result in inequitable treatment decisions and worsen existing healthcare disparities [13].

A potential source of algorithmic unfairness is sampling bias where the algorithm performs inferiorly in subgroups of patients that are underrepresented in the training data. Although registries such as the National Cancer Database (NCDB) offer an opportunity to include larger samples of minority populations, the dataset remains skewed toward the majority demographic due to the composition of contributing institutions [14]. As a result, models trained on such datasets may inadvertently favor White patients while underperforming for underrepresented groups such as Black, Hispanic, or Asian patients [15]. This is particularly concerning in prostate cancer where Black patients are disproportionately affected by aggressive disease and poor outcomes [16].

To support equitable use of predictive models in prostate cancer care, it is essential to evaluate algorithm performance across diverse race subgroups and to adopt strategies that promote fairness in model development [6, 17, 18, 19]. Recent methodological research has made significant strides in defining fairness for time‐to‐event data, proposing techniques ranging from regularizers that enforce hazard parity [20, 21] and mutual information minimization [22, 23] to distributionally robust optimization objectives [24]. However, these innovations have primarily served as methodological demonstrations on benchmark datasets, with limited translation to specific clinical domains such as prostate cancer. A previous study was unsuccessful at mitigating racial disparities in predicting survival after RP and considered this task as a binary classification problem [9]. Here, we extend this work by addressing the challenge as a survival analysis problem and directly compare two fairness‐aware approaches to mitigate subgroup performance disparities. Methodologically, we adapted Group Distributionally Robust Optimization (GroupDRO) to the survival setting particularly tailored to the Cox proportional hazards framework by formulating a worst‐case objective based on the Cox model's partial log‐likelihood, which is distinct from the methodology of [24]. Our goal was to improve the equity and clinical utility of ML‐based prognostication in prostate cancer by advancing methods for fair survival prediction.

## 2 Methods

### 2.1 Data Source

To develop our prediction model, we used data from the NCDB, a hospital‐based clinical cancer registry jointly managed by the American College of Surgeons Commission on Cancer and the American Cancer Society. The database captures over 70% of newly diagnosed cancers in the United States, making it one of the most comprehensive sources of oncology data for population‐level research [25]. The registry includes detailed clinical, demographic, and treatment‐related variables, which are critical for constructing a risk prediction model that accounts for both biological and social factors. The standardized data collection process used by Commission on Cancer‐accredited facilities helps ensure consistency, which is essential for building reliable predictive models. We utilized the 2021 NCDB participant user file. We note that this study was exempted by the NYU Langone Health institutional review board committee.

### 2.2 Cohort Selection and Stratification on Race/Ethnicity

We identified patients with clinically localized (cT1‐4/N0X/M0X), histologically confirmed invasive prostate cancer that underwent primary RP. We excluded patients with incomplete follow‐up data and those with missing pathological stage, pathological grade, or PSA.

We stratified patients into five groups using the National Institutes of Health and United States Office of Management and Budget race categories [26]: White, Black, Asian, Hispanic, and Other.

### 2.3 Predictor Variables

We adopted preprocessing steps consistent with those described in [9] for the NCDB dataset. We included demographic characteristics (age, race, insurance status, regional household income quartile, regional educational attainment, distance to facility, and urban/rural status) and clinical information (year of diagnosis, pre‐treatment PSA level, pathological tumor stage, grade group at RP, and Charlson/Deyo comorbidity score), and provider characteristics (facility type, facility location, and whether more than one facility submitted a report for the case to the NCDB) as predictor variables in our survival models. We required complete data on critical diagnostic features for study inclusion; consequently, patients missing information essential for defining clinical outcomes (e.g., pathological stage, pathological grade, or PSA) were excluded during the cohort selection procedure. As a result, the remaining continuous variables were complete without the need for imputation. For the remaining categorical covariates with missingness, missing values were modeled as a distinct category. For predictors that were reported in multiple years (e.g., regional education attainment in years 2000, 2012, 2016, and 2020), we included all years as separate predictors. We further note that we did not include the sensitive variable, that is, race/ethnicity, as a predictor variable.

### 2.4 Outcome

The primary outcome was overall survival, which accounts for mortality from any cause. Survival time was measured from the date of treatment until the last follow‐up or death, as recorded in the NCDB.

### 2.5 Statistical Analysis

We employed a deep learning‐based Cox proportional hazards model to predict overall survival after RP. The Cox proportional hazard framework is well‐suited for this task due to its ability to produce continuous risk scores that support clinically meaningful risk stratification. The proportional hazards structure also facilitates model interpretability, an important factor for clinical adoption. Moreover, Cox‐based models naturally handle censoring and provide time‐to‐event predictions, offering more clinically relevant insights than binary classification methods [27].

#### 2.5.1 Baseline Survival Model

We used the Deep Cox Proportional Hazards (DCPH) model as our baseline, which extends the traditional Cox model by allowing nonlinear relationships between covariates and the hazard function while preserving the familiar hazard ratio framework. We refer to this baseline as the baseline DCPH. The baseline DCPH was trained with the following loss function:

$$L_{\text{Base}}\left(\theta \right)=-\frac{1}{n}\sum_{i:\delta_{i}=1}\left(f_{\theta }\left(x_{i}\right)-\log\sum_{j\in R\left(o_{i}\right)}\exp\;f_{\theta }\left(x_{j}\right)\right),$$

where n is the number of patients whose events were observed, $f_{\theta }\left(x_{i}\right)$ is the log‐hazard predicted by the DCPH and $R\left(o_{i}\right)$ is the risk set at time $o_{i}$.

#### 2.5.2 Fairness‐Aware Survival Models

While the baseline DCPH improves survival prediction by capturing complex, nonlinear relationships, it does not account for fairness and may result in prediction performance disparities. To mitigate these issues, we employ two distinct fairness‐aware approaches: (1) Fair DCPH [20], an extension to DCPH that explicitly integrates fairness constraints into the loss function to balance hazard estimation across a pre‐specified protected attribute, such as race groups, whose loss function is:

$$L_{\text{Fair}}\left(\theta \right)=L_{\text{Base}}\left(\theta \right)+\sum_{k=1}^{K}{\left({\bar{f}}_{\theta ,k}-{\bar{f}}_{\theta }\right)}^{2},$$

where ${\bar{f}}_{\theta ,k}$ and ${\bar{f}}_{\theta }$ are the average estimated log‐hazard for group k and the average estimate log‐hazard for the entire data; and (2) Group Distributionally Robust Optimization (GroupDRO) DCPH [28], which optimizes worst‐case group performance across a pre‐specified protected attribute. We adapted GroupDRO to DCPH by maximizing the worst case negative partial log‐likelihood:

$$L_{\text{GroupDRO}}\left(\theta \right)=\max_{k=0,1,\ldots ,K}-\frac{1}{\left|n_{k}\right|}\sum_{i:\delta_{i}=1,s_{i}=k}\left(f_{\theta }\left(x_{i}\right)-\log\sum_{j\in R\left(o_{i}\right)}\exp\;f_{\theta }\left(x_{j}\right)\right),$$

where $n_{k}$ is the number of individuals with observed events in group k and $R\left(o_{i}\right)$ is the risk set at time $o_{i}$. We emphasize that the risk set is constructed over the entire cohort and is not group‐specific. Finally, the optimization includes an augmented group k=0 representing the full cohort. This ensures that the global fit is maintained as a baseline, preventing overall performance degradation while optimizing for the worst‐case subgroup.

#### 2.5.3 Baseline Hazard Estimation

Following the optimization of the deep learning parameters θ, we estimate the nonparametric baseline hazard function, $h_{0}\left(t\right)$, using the Breslow estimator [29]. This post hoc step is required to convert the partial risk scores produced by the deep learning into absolute survival probabilities. For each unique event time $t_{j}$, the discrete baseline hazard contribution is computed as the ratio of the number of observed events at that time to the total accumulated risk of the individuals remaining in the risk set. Specifically, let $d_{j}$ be the number of events at time $t_{j}$, and let $R\left(t_{j}\right)$ be the set of individuals at risk at time $t_{j}$. The baseline hazard at $t_{j}$ is calculated by:

$$h_{0}\left(t_{j}\right)=\frac{d_{j}}{\sum_{l\in R\left(t_{j}\right)}\exp f_{\theta }\left(x_{l}\right)}.$$

Therefore, the predicted survival probability of a patient $x_{i}$ at time t is calculated by:

$${\hat{S}}_{l}\left(t\right)=\exp\left(-\left[\sum_{j:t_{j}\leq t}h_{0}\left(t_{j}\right)\right]\times \exp\;f_{\theta }\left(x_{i}\right)\right).$$

#### 2.5.4 Deep Learning Architectures and Training Details

We implemented a deep neural network based on the Cox Proportional Hazards framework. The architecture consists of a Multi‐Layer Perceptron (MLP) designed to estimate the log‐hazard ratio for a given input feature vector. The network is composed of an input linear layer mapping the feature dimension to a hidden dimension of 32. This is followed by a sequential backbone consisting of four fully connected (dense) layers. The hidden layers maintain a width of 32 neurons and utilize Scaled Exponential Linear Unit (SELU) activations to induce self‐normalizing properties during training. The final layer projects the hidden representation to a single scalar output representing the predicted risk score. No batch normalization or dropout was applied.

We employed a train‐validation split (80% training, 20% validation), stratified by event status to ensure balanced event distribution. We optimized the network parameters using Stochastic Gradient Descent (SGD) with a momentum of 0.9 and an initial learning rate of 0.01. The learning rate was modulated using a step scheduler, decaying by a factor of 0.1 every 5 epochs, while weight decay was applied with a coefficient of 0.001. Models were trained with a batch size of 128 for a maximum of 100 epochs. To mitigate overfitting, we employed an early stopping mechanism that monitored validation loss with a patience of 5 epochs. Finally, random seeds for the framework and data splitting were fixed, respectively, to ensure reproducibility.

#### 2.5.5 Statistical Softwares and Computational Environments

All experiments were performed on a PC equipped with an Intel Core i7‐13700K processor and 32 GB of system RAM. The software environment was based on Python 3.9, with deep learning models implemented in PyTorch 2.0.0. Model training was accelerated using a single NVIDIA GeForce RTX 4070 GPU, utilizing CUDA 11.8 and cuDNN 8.7.0.

#### 2.5.6 Evaluation Metrics

We use Harrell's concordance index (C‐index) [30] to evaluate the discrimination performance of survival models. The C‐index quantifies this ability by measuring the concordance between predicted risks and observed survival times, accounting for censoring. Specifically, it estimates the probability that, for a randomly selected pair of individuals, the patient with the higher predicted risk experiences the event sooner.

In addition to overall discrimination, we evaluated the model's performance within race subgroups using cross‐group and within‐group C‐indices. We estimated 95% confidence intervals (CI) using bootstrapping with 1000 bootstrap samples.

Cross‐group C‐index evaluates how well the model differentiates individuals from a specific subgroup relative to others in the dataset in any group. Specifically, it includes all comparable pairs where at least one individual belongs to the group of interest. This captures whether the model consistently assigns higher risk to patients who truly experience events earlier, even when those patients belong to different subgroups. It is useful for detecting systematic biases and assessing fairness in relative risk predictions across groups. Cross‐group C‐index is defined as:

$$C_{k}^{\text{cross}}=\frac{\sum_{\left(i.j\right)\in \Omega_{k}^{\text{cross}}}\left(I\left(r_{i}>r_{j}\right)+0.5I\left(r_{i}=r_{j}\right)\right)}{|\Omega_{k}^{\text{cross}}|},$$

where $\Omega_{k}^{\text{cross}}=\left\{\left(i,j\right):\left(o_{i}<o_{j}\right)\wedge \left(\delta_{i}=1\right)\wedge \left(s_{i}=k\wedge s_{j}\neq k\right)\vee \left(s_{i}\neq k\wedge s_{j}=k\right)\right\}$ is the set of inter‐group comparable pairs associated with group k, $o_{i}$ is the observed follow‐up time, $\delta_{i}$ is the event indicator, $s_{i}$ is the sensitive attribute, and $r_{i}$ is predicted risk of individual i.

Within‐group C‐index evaluates how well a model differentiates individuals within the same subgroup, and it assesses only intragroup ranking, ignoring how well the model differentiates risk across subgroups. This can mask clinically important disparities. For instance, a model that correctly assigns higher risk to Black patients than to White patients (in line with the true hazard structure) would not be considered by within‐group evaluations. It is defined as:

$$C_{k}^{\text{within}}=\frac{\sum_{\left(i.j\right)\in \Omega_{k}}\left(I\left(r_{i}>r_{j}\right)+0.5I\left(r_{i}=r_{j}\right)\right)}{|\Omega_{k}|},$$

where $\Omega_{k}=\left\{\left(i,j\right):\left(o_{i}<o_{j}\right)\wedge \left(\delta_{i}=1\right)\wedge \left(s_{i}=s_{j}=k\right)\right\}$ is the set of comparable pairs associated with group k.

Given these differences, we primarily focus on the cross‐group C‐index. While within‐group metrics remain informative, the cross‐group measure better reflects the model's fairness by assessing whether it preserves meaningful risk stratification across subgroups.

Moreover, we consider integrated Brier score (IBS) and integrated calibration index (ICI), which measure calibration performances of survival analysis models. IBS for group k is calculated by integrating the Brier Score (mean squared error between predicted probabilities and observed status) over the evaluation time horizon, adjusted for censoring via inverse probability weighting. It provides an aggregate measure of prediction accuracy. IBS for group k is defined as:

${\mathrm{IBS}}_{k}=\frac{1}{\tau }\int_{0}^{\tau }\left(\frac{1}{\left|\Omega_{k}\right|}\sum_{i\in \Omega_{k}}w_{i}\left(t\right){\left(I\left(o_{i}>t\right)-{\hat{S}}_{i}\left(t\right)\right)}^{2}\right)\mathrm{dt},$where τ is the maximum follow‐up time, $\hat{P}\left(T>t|{\hat{S}}_{i}\left(t\right)\right)$ is the predicted survival probability for individual i at time t, and $w_{i}\left(t\right)$ is the inverse probability of censoring weight. Lower IBS values indicate better calibration and accuracy. The ICI evaluates how closely the predicted survival probabilities align with the observed event rates across the range of predicted risks. It is calculated as the mean absolute difference between the predicted survival probability and the calibrated survival probability (estimated using a flexible regression method, such as a loess smoother) integrated over time. ICI for group k is defined as:

$$\mathrm{IC}I_{k}=\frac{1}{\tau }\int_{0}^{\tau }E\left[\left|{\hat{S}}_{i}\left(t\right)-\hat{P}\left(T>t|{\hat{S}}_{i}\left(t\right)\right)\right||s_{i}=k\;\right]\mathrm{dt},$$

where $\hat{P}\left(T>t|{\hat{S}}_{i}\left(t\right)\right)$ represents the observed survival probability derived from the calibration curve for the predicted survival probability ${\hat{S}}_{i}\left(t\right)$. Lower ICI values indicate better calibration, implying that the predicted risks accurately reflect the true probability of survival.

#### 2.5.7 Risk‐Stratification by Race Group

Although the C‐index is a widely used metric to assess discrimination performance, it does not capture whether a model systematically overestimates or underestimates risks across different groups [31]. To further investigate these disparities, we stratified patients into three risk categories based on the model's predicted risks: high risk (top 25%), mid risk (25%–75%), and low risk (bottom 25%). We then compared the observed and predicted survival curves within each group using two‐sided pairwise log‐rank tests at a significance level of 0.05 [32].

## 3 Results

### 3.1 Cohort Characteristics

We identified 2,161,253 patients with a primary site diagnosis of prostate cancer between 2004 and 2021. Of these, 418,968 met inclusion criteria (Figure S1). The majority (78.5%, *n* = 328,937) of the population was classified as White, followed by Black (13.2%, *n* = 55,275), Hispanic (4.5%, *n* = 18,648), Other (2.0%, *n* = 8262), and Asian (1.9%, *n* = 7846). Their characteristics are shown in Table 1. Black patients were diagnosed at a younger age, most likely to belong to the lowest income quartile, and had a higher comorbidity burden relative to other races. White patients were more likely to be evaluated in community cancer programs compared to other races. Pathologic tumor stage and grade also showed variation, with Black patients presenting with higher‐grade disease more frequently compared to White patients.

**TABLE 1:** Baseline characteristics of patients undergoing radical prostatectomy for clinically localized prostate cancer.

	White (*n* = 328,937)	Black (*n* = 55,275)	Hispanic (*n* = 18,648)	Asian (*n* = 7846)	Other (*n* = 8262)
Age (median, IQR)	63.0 (58.0–67.0)	60.0 (55.0–65.0)	62.0 (56.0–66.0)	64.0 (59.0–68.0)	62.0 (56.0–66.0)
Year of diagnosis (median, IQR)	2015 (2012–2018)	2015 (2012–2018)	2016 (2013–2018)	2016 (2012–2018)	2015 (2012–2018)
Facility type (*n*, %)					
Community Cancer Program	13,006 (3.95%)	1506 (2.72%)	579 (3.10%)	147 (1.87%)	259 (3.13%)
Comprehensive Community Cancer Program	117,168 (35.62%)	17,209 (31.13%)	5622 (30.15%)	1858 (23.68%)	2253 (27.27%)
Academic/Research Program	133,591 (40.61%)	25,445 (46.03%)	9521 (51.06%)	4595 (58.56%)	4081 (49.39%)
Integrated Network Cancer Program	64,980 (19.75%)	11,031 (19.96%)	2909 (15.60%)	1241 (15.82%)	1660 (20.09%)
Unknown	192 (0.06%)	84 (0.15%)	17 (0.09%)	5 (0.06%)	9 (0.11%)
Facility location (*n*, %)					
New England	20,313 (6.18%)	1572 (2.84%)	970 (5.20%)	260 (3.31%)	553 (6.69%)
Middle Atlantic	46,641 (14.18%)	7792 (14.10%)	3332 (17.87%)	1425 (18.16%)	1707 (20.66%)
South Atlantic	59,319 (18.03%)	19,567 (35.40%)	3727 (19.99%)	892 (11.37%)	1607 (19.45%)
East North Central	60,584 (18.42%)	8311 (15.04%)	1495 (8.02%)	734 (9.36%)	1137 (13.76%)
East South Central	27,290 (8.30%)	6721 (12.16%)	202 (1.08%)	99 (1.26%)	160 (1.94%)
West North Central	38,881 (11.82%)	2367 (4.28%)	394 (2.11%)	212 (2.70%)	771 (9.33%)
West South Central	20,831 (6.33%)	5682 (10.28%)	3007 (16.13%)	459 (5.85%)	712 (8.62%)
Mountain	17,899 (5.44%)	519 (0.94%)	1131 (6.06%)	171 (2.18%)	492 (5.95%)
Pacific	36,987 (11.24%)	2660 (4.81%)	4373 (23.45%)	3589 (45.74%)	1114 (13.48%)
Not available	192 (0.06%)	84 (0.15%)	17 (0.09%)	5 (0.06%)	9 (0.11%)
Insurance status (*n*, %)					
Not insured	2652 (0.81%)	1430 (2.59%)	772 (4.14%)	128 (1.63%)	148 (1.79%)
Private insurance/managed care	194,211 (59.04%)	32,186 (58.23%)	10,313 (55.30%)	4472 (57.00%)	4964 (60.08%)
Medicaid	5009 (1.52%)	3729 (6.75%)	1509 (8.09%)	409 (5.21%)	373 (4.51%)
Medicare	118,505 (36.03%)	15,185 (27.47%)	5624 (30.16%)	2701 (34.43%)	2416 (29.24%)
Other government insurance	5863 (1.78%)	2200 (3.98%)	237 (1.27%)	53 (0.68%)	241 (2.92%)
Unknown	2697 (0.82%)	545 (0.99%)	193 (1.03%)	83 (1.06%)	120 (1.45%)
Median income quartile 2000 (*n*, %)					
< $30,000	20,276 (6.16%)	11,320 (20.48%)	2954 (15.84%)	232 (2.96%)	764 (9.25%)
$30,000–$34,999	41,347 (12.57%)	8122 (14.69%)	2562 (13.74%)	456 (5.81%)	914 (11.06%)
$35,000–$45,999	75,667 (23.00%)	11,942 (21.60%)	4290 (23.01%)	1171 (14.92%)	1684 (20.38%)
≥ $46,000	138,831 (42.21%)	15,037 (27.20%)	6122 (32.83%)	4796 (61.13%)	3637 (44.02%)
Unknown	52,816 (16.06%)	8854 (16.02%)	2720 (14.59%)	1191 (15.18%)	1263 (15.29%)
Median income quartile 2012 (*n*, %)					
< $38,000	28,340 (8.62%)	15,572 (28.17%)	3574 (19.17%)	320 (4.08%)	906 (10.97%)
$38,000–$47,999	58,473 (17.78%)	10,393 (18.80%)	3576 (19.18%)	645 (8.22%)	1274 (15.42%)
$48,000–$62,999	80,112 (24.35%)	10,653 (19.27%)	4489 (24.07%)	1496 (19.07%)	1919 (23.23%)
≥ $63,000	117,882 (35.84%)	11,091 (20.07%)	4810 (25.79%)	4416 (56.28%)	3172 (38.39%)
Unknown	44,130 (13.42%)	7566 (13.69%)	2199 (11.79%)	969 (12.35%)	991 (11.99%)
Median income quartile 2016 (*n*, %)					
< $40,227	29,630 (9.01%)	16,376 (29.63%)	3759 (20.16%)	354 (4.51%)	952 (11.52%)
$40,227–$50,353	55,615 (16.91%)	9916 (17.94%)	3440 (18.45%)	624 (7.95%)	1194 (14.45%)
$50,354–$63,332	69,512 (21.13%)	8617 (15.59%)	3766 (20.20%)	1214 (15.47%)	1616 (19.56%)
≥ $63,333	127,061 (38.63%)	12,124 (21.93%)	5283 (28.33%)	4641 (59.15%)	3425 (41.45%)
Unknown	47,119 (14.32%)	8242 (14.91%)	2400 (12.87%)	1013 (12.91%)	1075 (13.01%)
Median income quartile 2020 (*n*, %)					
< $46,277	27,799 (8.45%)	15,636 (28.29%)	3362 (18.03%)	280 (3.57%)	882 (10.68%)
$46,277–$57,856	56,238 (17.10%)	10,435 (18.88%)	3276 (17.57%)	567 (7.23%)	1220 (14.77%)
$57,857–$74,062	71,026 (21.59%)	9029 (16.33%)	4098 (21.98%)	1238 (15.78%)	1659 (20.08%)
≥ $74,063	126,393 (38.42%)	11,906 (21.54%)	5507 (29.53%)	4747 (60.50%)	3425 (41.45%)
Unknown	47,481 (14.43%)	8269 (14.96%)	2405 (12.90%)	1014 (12.92%)	1076 (13.02%)
Regional education attainment 2000 (*n*, %)					
≥ 29.0%	25,023 (7.61%)	13,008 (23.53%)	5867 (31.46%)	685 (8.73%)	890 (10.77%)
20.0%–28.9%	51,216 (15.57%)	12,675 (22.93%)	3436 (18.43%)	1098 (13.99%)	1333 (16.13%)
14.0%–19.9%	68,275 (20.76%)	9132 (16.52%)	2659 (14.26%)	1204 (15.35%)	1577 (19.09%)
< 14.0%	131,586 (40.00%)	11,598 (20.98%)	3960 (21.24%)	3668 (46.75%)	3198 (38.71%)
Unknown	52,837 (16.06%)	8862 (16.03%)	2726 (14.62%)	1191 (15.18%)	1264 (15.30%)
Regional education attainment 2012 (*n*, %)					
≥ 21.0%	25,410 (7.72%)	12,744 (23.06%)	6665 (35.74%)	991 (12.63%)	971 (11.75%)
13.0%–20.9%	57,720 (17.55%)	15,645 (28.30%)	3845 (20.62%)	1236 (15.75%)	1511 (18.29%)
7.0%–12.9%	101,540 (30.87%)	12,856 (23.26%)	3619 (19.41%)	2157 (27.49%)	2425 (29.35%)
< 7.0%	100,263 (30.48%)	6494 (11.75%)	2326 (12.47%)	2494 (31.79%)	2372 (28.71%)
Unknown	44,004 (13.38%)	7536 (13.63%)	2193 (11.76%)	968 (12.34%)	983 (11.90%)
Regional education attainment 2016 (*n*, %)					
≥ 17.6%	31,867 (9.69%)	15,116 (27.35%)	7437 (39.88%)	1226 (15.63%)	1128 (13.65%)
10.9%–17.5%	60,978 (18.54%)	14,572 (26.36%)	3509 (18.82%)	1185 (15.10%)	1597 (19.33%)
6.3%–10.8%	88,104 (26.78%)	11,114 (20.11%)	2992 (16.04%)	2006 (25.57%)	2112 (25.56%)
< 6.3%	101,328 (30.80%)	6296 (11.39%)	2330 (12.49%)	2419 (30.83%)	2364 (28.61%)
Unknown	46,660 (14.19%)	8177 (14.79%)	2380 (12.76%)	1010 (12.87%)	1061 (12.84%)
Regional education attainment 2020 (*n*, %)					
≥ 15.3%	32,567 (9.90%)	14,701 (26.60%)	7714 (41.37%)	1312 (16.72%)	1232 (14.91%)
9.1%–15.2%	67,086 (20.39%)	15,966 (28.88%)	3761 (20.17%)	1426 (18.17%)	1723 (20.85%)
5.0%–9.0%	93,727 (28.49%)	11,279 (20.41%)	2834 (15.20%)	2004 (25.54%)	2164 (26.19%)
< 5.0%	88,897 (27.03%)	5149 (9.32%)	1960 (10.51%)	2094 (26.69%)	2083 (25.21%)
Unknown	46,660 (14.19%)	8180 (14.80%)	2379 (12.76%)	1010 (12.87%)	1060 (12.83%)
Rurality and urban influence 2003 (*n*, %)					
Counties in metro areas of 1 million population or more	145,555 (44.25%)	32,972 (59.65%)	12,552 (67.31%)	5430 (69.21%)	4219 (51.07%)
Counties in metro areas of 250,000 to 1 million population	68,723 (20.89%)	10,588 (19.16%)	3617 (19.40%)	1588 (20.24%)	1574 (19.05%)
Counties in metro areas of fewer than 250,000 population	36,658 (11.14%)	4326 (7.83%)	996 (5.34%)	233 (2.97%)	662 (8.01%)
Urban population of 2500–19,999, adjacent to a metro area	18,617 (5.66%)	1719 (3.11%)	354 (1.90%)	62 (0.79%)	418 (5.06%)
Urban population of 20,000 or more adjacent to a metro area	5957 (1.81%)	481 (0.87%)	164 (0.88%)	172 (2.19%)	206 (2.49%)
Urban population of 2500–19,999, not adjacent to a metro area	8630 (5.66%)	1940 (3.51%)	266 (1.43%)	28 (0.36%)	268 (3.24%)
Urban population of 20,000 or more not adjacent to a metro area	9782 (2.97%)	658 (1.19%)	151 (0.81%)	17 (0.22%)	185 (2.24%)
Completely rural or less than 2500 urban population, not adjacent to a metro area	3507 (1.07%)	314 (0.57%)	22 (0.12%)	6 (0.08%)	51 (0.62%)
Completely rural or less than 2500 urban population, adjacent to a metro area	3888 (1.18%)	296 (0.54%)	28 (0.15%)	5 (0.06%)	102 (1.23%)
Not available	17,620 (5.36%)	1981 (3.58%)	498 (2.67%)	305 (3.89%)	577 (6.98%)
Rurality and urban influence 2013 (*n*, %)					
Counties in metro areas of 1 million population or more	149,694 (45.51%)	33,609 (60.80%)	12,699 (68.10%)	5481 (69.86%)	4349 (52.64%)
Counties in metro areas of 250,000 to 1 million population	72,883 (22.16%)	11,126 (20.13%)	3820 (20.48%)	1614 (20.57%)	1632 (19.75%)
Counties in metro areas of fewer than 250,000 population	34,012 (10.34%)	3877 (7.01%)	739 (3.96%)	200 (2.55%)	596 (7.21%)
Urban population of 2500–19,999, adjacent to a metro area	16,390 (4.98%)	1344 (2.43%)	299 (1.60%)	52 (0.66%)	360 (4.36%)
Urban population of 20,000 or more adjacent to a metro area	5027 (1.53%)	383 (0.69%)	153 (0.82%)	148 (1.89%)	162 (1.96%)
Urban population of 2500–19,999, not adjacent to a metro area	17,740 (5.39%)	1762 (3.19%)	246 (1.32%)	24 (0.31%)	255 (3.09%)
Urban population of 20,000 or more not adjacent to a metro area	9233 (2.81%)	580 (1.05%)	150 (0.80%)	10 (0.13%)	189 (2.29%)
Completely rural or less than 2500 urban population, not adjacent to a metro area	2811 (0.85%)	380 (0.69%)	18 (0.10%)	7 (0.09%)	39 (0.47%)
Completely rural or less than 2500 urban population, adjacent to a metro area	3523 (1.07%)	233 (0.42%)	26 (0.14%)	5 (0.06%)	102 (1.23%)
Not available	17,624 (5.36%)	1981 (3.58%)	498 (2.67%)	305 (3.89%)	578 (7.00%)
Distance to facility (median (IQR))	21.1 (8.4–74.9)	12.2 (5.5–43.4)	11.7 (5.2–35.12)	11.8 (5.7–34.0)	20.9 (8.0–90.5)
More than one CoC accredited facility submitted a report for the case to the NCDB (*n*, %)					
No	290,120 (88.20%)	49,449 (89.46%)	17,130 (91.86%)	7019 (89.46%)	7368 (89.18%)
Yes	38,817 (11.80%)	5826 (10.54%)	1518 (8.14%)	827 (10.54%)	894 (10.82%)
Comorbidity score (Charlson‐Deyo Score) (*n*, %)					
0	271,950 (82.68%)	41,585 (75.23%)	14,958 (80.21%)	6151 (78.40%)	6762 (81.84%)
1	45,663 (13.88%)	10,189 (18.43%)	2959 (15.87%)	1350 (17.21%)	1215 (14.71%)
2	8114 (2.47%)	2115 (3.83%)	455 (2.44%)	231 (2.94%)	205 (2.48%)
3+	3210 (0.98%)	1386 (2.51%)	276 (1.48%)	114 (1.45%)	80 (0.97%)
Pre‐treatment PSA (median (IQR))	5.8 (4.5–8.3)	6.5 (4.8–10.0)	6.4 (4.8–9.6)	6.7 (5.0–9.9)	6.0 (4.6–8.8)
Posttreatment tumor grade (*n*, %)					
1	58,869 (17.90%)	8911 (16.12%)	3337 (17.89%)	1003 (12.78%)	1448 (17.53%)
2	157,408 (47.85%)	27,629 (49.98%)	8608 (46.16%)	3525 (44.93%)	3933 (47.60%)
3	68,779 (20.91%)	11,845 (21.43%)	3908 (20.96%)	1944 (24.78%)	1800 (21.79%)
4	21,124 (6.42%)	3529 (6.38%)	1397 (7.49%)	646 (8.23%)	527 (6.38%)
5	22,757 (6.92%)	3361 (6.08%)	1398 (7.50%)	728 (9.28%)	554 (6.71%)
Pathological tumor stage (*n*, %)					
T1/T2	231,165 (70.28%)	40,226 (72.77%)	12,870 (69.02%)	5292 (67.45%)	5718 (69.21%)
T3	97,348 (29.59%)	14,959 (27.06%)	5729 (30.72%)	2539 (32.36%)	2537 (30.71%)
T4	424 (0.13%)	90 (0.16%)	49 (0.26%)	15 (0.19%)	7 (0.08%)

### 3.2 Cross‐Group Fairness Evaluation

Table 2 presents the cross‐group C‐indices for three variants of the DCPH model, Baseline DCPH, Fair DCPH, and GroupDRO DCPH, evaluated across race subgroups. The baseline DCPH achieves the highest performance for the White subgroup (C‐index = 0.699 [95% CI 0.695–0.703]). In contrast, the baseline DCPH performs substantially worse for less represented groups such as Black (C‐index = 0.678 [95% CI 0.671–0.685]) and Hispanic patients (C‐index = 0.689 [95% CI 0.684–0.694]), indicating unfairness in discrimination performance. Both the Fair DCPH and GroupDRO DCPH improve the cross‐group C‐index for these underrepresented groups. For the Black subgroup, the C‐index increases to 0.692 (95% CI 0.686–0.698) (Fair DCPH) and 0.696 (95% CI 0.691–0.701) (GroupDRO DCPH), and for the Hispanic subgroup, it increases to 0.693 (95% CI 0.688–0.698) and 0.697 (0.693–0.701), respectively. These improvements are achieved with only a modest reduction in performance for the White subgroup and a slight decrease in the overall C‐index (from 0.694 [95% CI 0.691–0.697] in the baseline DCPH to 0.688 [95% CI 0.684–0.692] and 0.689 [95% CI 0.686–0.692] in the fairness‐aware models).

**TABLE 2:** Comparison of the baseline and fairness‐aware DCPH models.

Group	Sample size (*n*)	Baseline DCPH				Fair DCPH				GroupDRO DCPH			
		Within‐group C‐index	Cross‐group C‐index	IBS	ICI	Within‐group C‐index	Cross‐group C‐index	IBS	ICI	Within‐group C‐index	Cross‐group C‐index	IBS	ICI
Overall	418,968	**0.694** (0.691–0.697)	—	**0.287** (0.284–0.289)	**0.437** (0.435–0.438)	**0.688** (0.684–0.692)	—	**0.286** (0.284–0.289)	**0.437** (0.435–0.439)	**0.689** (0.686–0.692)	—	**0.287** (0.285–0.290)	**0.427** (0.425–0.429)
White	328,937	**0.690** (0.686–0.694)	**0.699** (0.695–0.703)	**0.281** (0.278–0.283)	**0.429** (0.427–0.431)	**0.688** (0.684–0.692)	**0.689** (0.685–0.693)	**0.285** (0.283–0.288)	**0.429** (0.427–0.431)	**0.688** (0.684–0.692)	**0.693** (0.690–0.696)	**0.299** (0.297–0.301)	**0.439** (0.437–0.441)
Black	55,275	**0.681** (0.676–0.686)	**0.678** (0.671–0.685)	**0.367** (0.361–0.374)	**0.518** (0.513–0.523)	**0.691** (0.685–0.697)	**0.692** (0.686–0.698)	**0.311** (0.306–0.317)	**0.468** (0.463–0.472)	**0.693** (0.686–0.700)	**0.696** (0.691–0.701)	**0.197** (0.193–0.202)	**0.299** (0.293–0.304)
Hispanic	18,648	**0.685** (0.679–0.691)	**0.689** (0.684–0.694)	**0.218** (0.211–0.228)	**0.385** (0.378–0.392)	**0.686** (0.679–0.693)	**0.693** (0.688–0.698)	**0.282** (0.276–0.290)	**0.490** (0.484–0.495)	**0.687** (0.680–0.694)	**0.697** (0.693–0.701)	**0.206** (0.199–0.218)	**0.345** (0.338–0.353)
Asian	7846	**0.667** (0.661–0.673)	**0.696** (0.692–0.700)	**0.206** (0.195–0.220)	**0.363** (0.350–0.376)	**0.670** (0.664–0.676)	**0.702** (0.697–0.707)	**0.220** (0.214–0.229)	**0.431** (0.423–0.440)	**0.668** (0.661–0.675)	**0.707** (0.701–0.713)	**0.244** (0.231–0.258)	**0.413** (0.400–0.426)
Other	8262	**0.676** (0.670–0.682)	**0.686** (0.680–0.692)	**0.224** (0.213–0.233)	**0.379** (0.367–0.390)	**0.690** (0.684–0.696)	**0.698** (0.692–0.704)	**0.240** (0.236–0.244)	**0.441** (0.433–0.448)	**0.688** (0.681–0.695)	**0.692** (0.687–0.697)	**0.170** (0.161–0.179)	**0.293** (0.281–0.306)

*Note:* The numbers in the parentheses are 95% confidence interval estimated with bootstrapping.

### 3.3 Within‐Group Fairness Evaluation

To further examine model performance within each race subgroup, we also evaluated the within‐group C‐index. As shown in Table 2, the baseline DCPH model revealed a clear disparity in within‐group discrimination: while the White subgroup achieved the highest C‐index (0.690, 95% CI 0.686–0.694), the value steadily decreased for groups with smaller sample sizes, reaching the lowest levels in the Asian group at 0.667 (95% CI 0.661–0.673) and in Other at 0.676 (95% CI 0.670–0.682). This pattern suggests that the baseline model's discriminative ability diminishes as subgroup representation decreases. However, both the Fair DCPH and GroupDRO DCPH models substantially mitigated this disparity. In particular, the within‐group C‐index for the Black group improved to 0.691 (95% CI 0.685–0.697) with Fair DCPH and 0.693 (95% CI 0.686–0.700) with GroupDRO DCPH, and for Other, to 0.690 (95% CI 0.684–0.696) with Fair DCPH and 0.688 (95% CI 0.681–0.695) with GroupDRO DCPH. Performance for Asian and Hispanic subgroups also remained stable or improved slightly.

### 3.4 Integrated Brier Score and Integrated Calibration Index

Table 2 summarizes the IBSs and ICIs for the overall cohort and each race subgroup across the three models. Overall calibration performance was similar across models: in the full cohort, IBS remained essentially unchanged between the baseline DCPH (0.287, 95% CI 0.284–0.289) and the fairness‐aware models (0.286, 95% CI 0.284–0.289, for Fair DCPH and 0.287, 95% CI 0.285–0.290, for GroupDRO DCPH), while ICI was stable or slightly improved (0.437 for the baseline DCPH and Fair DCPH vs. 0.427 for GroupDRO DCPH). These findings indicate that incorporating fairness constraints did not meaningfully compromise global calibration.

At the subgroup level, the impact of fairness‐aware modeling on calibration was most notable for Black patients. For this group, IBS improved from 0.367 (95% CI 0.361–0.374) with the baseline DCPH to 0.311 (0.306–0.317) with Fair DCPH and 0.197 (0.193–0.202) with GroupDRO DCPH, accompanied by a reduction in ICI from 0.518 (0.513–0.523) to 0.468 (0.463–0.472) and 0.299 (0.293–0.304), respectively. GroupDRO DCPH also improved calibration for Hispanic and Other patients, with lower IBS and ICI compared with the baseline model, whereas Fair DCPH showed more variable effects in these smaller subgroups. In contrast, calibration for White and Asian patients was largely preserved, with only modest increases in IBS and ICI, consistent with the small trade‐offs in discrimination. Taken together, these results suggest that fairness‐aware survival models, particularly GroupDRO DCPH, can improve calibration for underrepresented race subgroups while maintaining acceptable overall calibration performance.

### 3.5 Risk‐Stratification by Race Group

**FIGURE 1:**
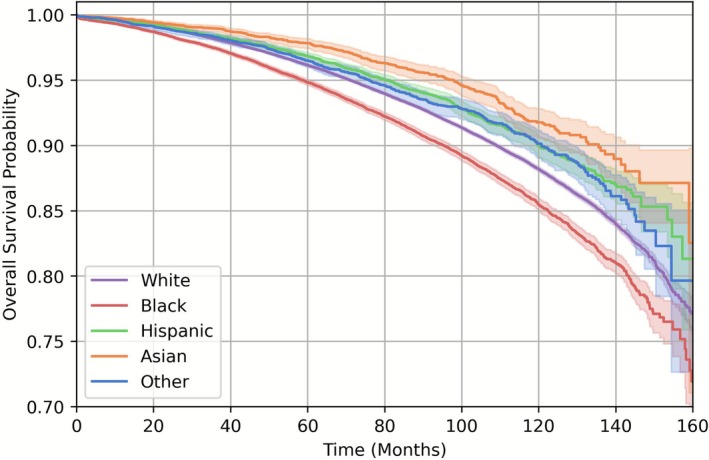
Observed survival curve per race. There are differences in survival probabilities across racial groups; notably, the survival probability for the Black subgroup is significantly lower compared to the White subgroup (*p* < 0.001; log‐rank test, two‐sided, *α* = 0.05).

The observed survival curves of each race/ethnicity group are presented in Figure 1. As visualized, the Black subgroup had a lower survival probability compared to the White subgroup. Meanwhile, race groups other than Black were more likely to survive compared to the White subgroup, which aligns with what has been reported by previous studies [33], including those from the NCDB [34]. This demonstrates that there exist differences in survival probabilities across race groups and thus if a risk prediction model is biased toward the White subgroup, the model will likely underestimate the risk for the Black subgroup; meanwhile, it can possibly overestimate the risk for Hispanic, Asian, and Other race groups.

**FIGURE 2:**
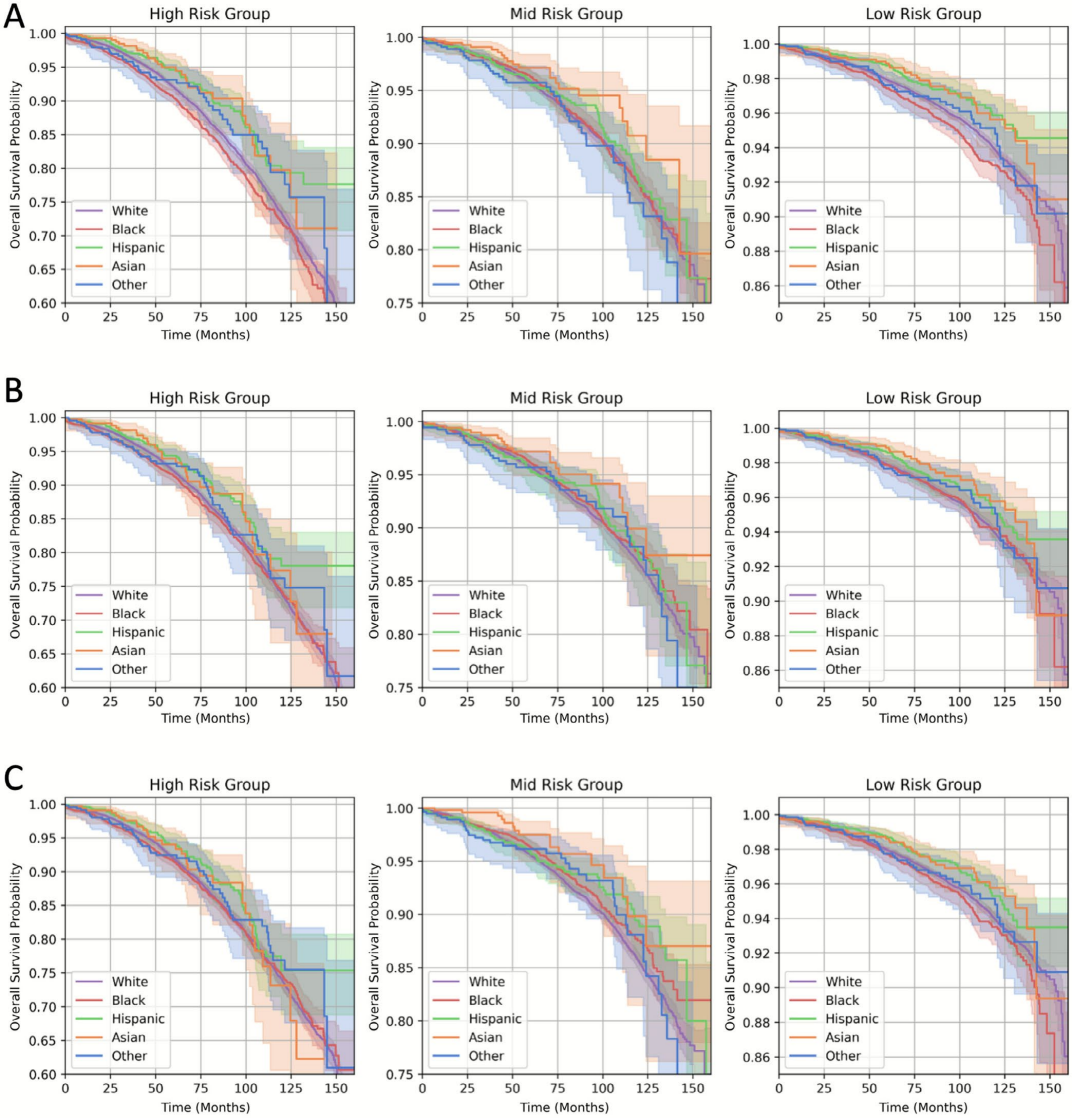
Observed survival curves for the three risk groups based on the baseline DCPH (A), Fair DCPH (B), and GroupDRO DCPH (C) models. Patients were stratified into high (top 25%), mid (25%–50%), and low (bottom 50%) risk categories. (A) The baseline DCPH model exhibits significant bias in the high‐risk category. The observed survival curve for the Black subgroup is significantly lower than other racial groups (*p* < 0.001; log‐rank test, two‐sided, *α* = 0.05), indicating the model underestimates their mortality risk. Conversely, the Hispanic subgroup shows significantly higher survival compared to the White group (*p* < 0.001), suggesting risk overestimation. (B, C) The Fair DCPH and GroupDRO DCPH models demonstrate the mitigation of disparity. The disparities for Black and Hispanic subgroups observed in the baseline are mitigated, resulting in nonsignificant differences across racial groups (*p* > 0.05) within the risk categories.

To further investigate the potential impact of the baseline and fair models, we stratified patients into three risk categories: high risk (top 25%), mid risk (25%–50%), and low risk (bottom 50%). Figure 2 presents the observed survival distributions across model predicted risk categories of high, mid, and low, stratified by Baseline (Figure 2A), Fair (Figure 2B), and GroupDRO DCPH (Figure 2C). In the presence of algorithmic fairness, survival curves should be similar across the protected attribute within each predicted risk group. As hypothesized, the baseline DCPH model, being biased toward the White subgroup, tended to underestimate risk for the Black subgroup while overestimating risk for Hispanic. In particular, for the high‐risk category, the observed survival curve for Black was substantially and significantly (*p* < 0.001; log‐rank test, two‐sided, *α* = 0.05) lower than that of other racial groups, indicating that the baseline DCPH model severely underestimates their risk of mortality especially for high‐risk group. Furthermore, the observed survival curve for Hispanic was significantly higher than that of White group (*p* < 0.001; log‐rank test, two‐sided, *α* = 0.05). In contrast, in the mid‐risk group, the observed survival curves did not show notable differences across race groups, suggesting that the model's predictions were relatively fair in this risk group. In the fairness‐aware survival distributions, the disparities in survival estimates for the Black and Hispanic subgroup were mitigated (*p* > 0.05; log‐rank test against all other racial groups, two‐sided, *α* = 0.05).

## 4 Discussion

Prediction models are being increasingly developed and applied in diverse settings, including healthcare, with many of the novel models being based on ML and AI‐based approaches. Although these approaches can augment predictive performance, there is growing concern about the potential for these models to demonstrate bias and drive disparities if they demonstrate algorithmic unfairness. In this study, we used an AI‐based approach to develop a model predicting survival after RP and demonstrated that a baseline model demonstrated algorithmic unfairness across race groups with superior predictive performance for White patients and worse performance for Black, Hispanic, and Asian patients. The potential for algorithmic unfairness is particularly concerning in prostate cancer, where Black patients already face disproportionately higher rates of aggressive disease and worse outcomes [33, 34, 35, 36, 37, 38]. To mitigate these disparities, we applied two distinct fairness‐aware modeling approaches, Fair DCPH and GroupDRO DCPH, which led to improved predictive performance for underrepresented race subgroups across multiple fairness metrics, including cross‐group and within‐group C‐indices, without substantial loss in overall model performance. Importantly, risk stratification analyses showed that fairness‐aware models more accurately aligned predicted and observed survival across race groups, supporting more equitable risk classification and reducing the likelihood of systematic under‐ or overestimation of risk based on race.

Estimating survival after RP is important to counsel patients, guide discussions about adjuvant therapy and intensity of follow‐up, and allocate supportive resources. For patients, understanding their predicted survival can shape expectations and enable more personalized planning. To this extent, developing a model for this clinical use has been a topic of interest in several previous studies [39, 40, 41, 42], including some that have been based on ML and AI‐based approaches [43, 44, 45, 46]. However, there has been limited investigation into the potential for algorithmic bias in this context [9]. The potential clinical impact of an unfair model is illustrated in Figure 2. For example, if the model was used to allocate resources, such as supportive care, to patients in the predicted high‐risk group, the baseline model may withhold resource allocation to high‐risk Black subgroup patients, despite their survival being worse than those of other race subgroups in the high‐risk group.

Given the growing adoption of prediction models, there has been increasing attention to algorithmic fairness. Early work on algorithmic fairness introduced criteria like demographic parity, equalized odds, and predictive parity to address bias in domains such as criminal justice and lending [47, 48, 49, 50]. As ML gained traction in healthcare, these fairness frameworks began to be adapted to clinical settings [13]. Despite growing interest in fairness in clinical ML, most existing research has focused on binary classification tasks, such as disease diagnosis or hospital admission prediction, where fairness metrics like equalized odds and demographic parity can be directly applied. In contrast, fairness in survival analysis has received considerably less attention. Survival models are central to many clinical applications, especially for long‐term prognostication, yet the adaptation of fairness frameworks to handle censored outcomes remains less explored [7, 51].

When developing prediction models, fairness‐aware approaches can be applied in the pre‐processing, in‐processing, or post‐processing phase [7]. Pre‐processing refers to data transformation before being used for model training. This was attempted in a prior study which sought to address sampling bias as a potential source of algorithmic unfairness in a model predicting survival after RP [9]. This study trained models in three scenarios: (1) a naturally race‐imbalanced sample, which represented the race proportions of the underlying population, (2) synthetically race‐balanced data, which had equal proportions of the races, and (3) a race‐homogeneous sample in which a separate model was trained for each race subgroup. This study found that these pre‐processing approaches were unsuccessful in improving model performance for underperforming race subgroups. In our study, we applied in‐processing fairness principles, which refer to approaches that incorporate fairness constraints during training. We found Fair and GroupDRO DCPH are alternative approaches to survival modeling that can successfully mitigate model unfairness; both approaches improved c‐metrics and survival plots which illustrated increased alignment of the race subgroups within each predicted risk group. In post‐processing approaches, algorithms transform the output of the model. However, an important limitation of post‐processing approaches is that the resulting model requires access to the protected attribute at the time of inference, which may not always be available [52].

It is critical to acknowledge the inherent trade‐off between maximizing overall predictive accuracy and ensuring algorithmic equity. Fairness‐aware training imposes constraints that can theoretically reduce the overall performance, as the algorithm is restricted from optimizing solely for the aggregate outcome. However, this potential trade‐off reflects a necessary ethical balance; accepting a marginal reduction in overall performance is often justifiable to ensure that prediction errors are not systematically concentrated within specific marginalized groups, thereby prioritizing equitable care over maximization of a single performance metric.

It is also important to distinguish the mechanisms by which these fairness‐aware approaches improve equity compared to traditional data‐level interventions. Unlike pre‐processing techniques that attempt to correct sampling bias through reweighting or synthetic oversampling, the methods employed here directly modify the optimization landscape. Fair DCPH mitigates disparity by incorporating a penalty term into the loss function that discourages the model from learning representations heavily correlated with the protected attribute, effectively regularizing the model to prioritize feature invariance. Conversely, GroupDRO DCPH operates by dynamically upweighting the contribution of the worst‐performing subgroup during training, ensuring that the optimization process is not dominated by the majority demographic. These mechanisms allow the models to regularize subgroup error directly, ensuring robust performance across populations without requiring the artificial alteration of the underlying data distribution. We selected Fair DCPH and GroupDRO DCPH because they provide explicit, tractable mechanisms for enforcing fairness constraints directly within the loss function of deep survival models tailored to the Cox proportional hazards model [20, 53]. Although alternative methods exist, such as reweighting [54, 55], adversarial debiasing [56, 57, 58], we note that while these approaches are well‐established for binary classification, their extension to right‐censored survival analysis is complex and less developed [24].

Beyond technical feasibility, there are regulatory, ethical, and interpretability considerations for deploying fairness‐aware models in clinical practice. Regulatory bodies such as the Federal Drug Agency are increasingly recognizing the importance of identifying and mitigating bias in clinical AI algorithms [59]. Ethically, it remains unknown what degree of unfairness would be considered acceptable for clinical use. From an interpretability perspective, providing end‐users with model explanations is necessary for translating technically fair models into tools that are safe and trustworthy. Practically, these fairness‐aware models could be incorporated into the electronic health record to streamline the workflow or deployed as an online tool for broader usability.

Our study has several strengths. First, we used a large, representative database of healthcare within the United States with comprehensive follow‐up data which allowed us to include large samples of the different race subgroups, as categorized by the National Institutes of Health and United States Office of Management and Budget race/ethnic categories [26]. Nonetheless, we recognize the heterogeneity within these groups and that fairness can be examined and optimized on other characteristics such as socioeconomic status or geographic location. Second, we compared baseline and fairness‐aware models on quantitative performance and also illustrated through risk groups how application of fairness‐unaware prediction models could deprive minority patients warranting additional resources from access, and how this could be mitigated by the application of fairness‐aware approaches. Finally, our study makes a novel methodological contribution by applying and comparing two fairness‐aware approaches in the context of survival analysis, a domain that has received far less attention than binary prediction tasks in fairness literature.

However, there are important limitations to our study. Although fairness‐aware modeling strategies improved performance equity across racial subgroups, complete parity was not achieved. Residual discrepancies in model discrimination and risk stratification persisted, likely reflecting structural inequities in the underlying data, differences in clinical presentation, and variation in access to care. Additionally, while the NCDB captures a significant proportion of nationwide annual cancer diagnoses, it is a hospital‐based registry susceptible to selection bias compared to population‐based registries. The reliance on area‐level socioeconomic proxies rather than individual‐level data also limits our ability to fully adjust for social determinants of health. Furthermore, although our models were developed and internally validated using a large, nationally representative dataset, external validation is required to understand the generalizability of applying these fairness‐aware approaches. Finally, we evaluated fairness based on race, but other sensitive attributes can be considered, such as insurance status or socioeconomic status. Despite these limitations, our study represents an important advance in developing fairness‐aware survival models and underscores the need to center equity in clinical prediction model design.

## 5 Conclusion

Our study demonstrates the feasibility and importance of integrating fairness‐aware techniques into survival prediction following RP. By improving model equity without compromising overall performance, these approaches offer a path forward for developing clinical decision support tools that promote equitable care.

## Author Contributions

**Madhur Nayan:** conceptualization, investigation, writing – original draft, writing – review and editing, methodology, visualization, data curation, supervision. **Rajesh Ranganath:** conceptualization, writing – review and editing. **Katie Murray:** conceptualization, writing – review and editing. **Hyungrok Do:** conceptualization, investigation, writing – original draft, methodology, validation, visualization, writing – review and editing, formal analysis, data curation.

## Ethics Statement

The study was exempted by the NYU Langone Health institutional review board committee.

## Conflicts of Interest

The authors declare no conflicts of interest.

## Supporting information


**Figure S1:** cam471544‐sup‐0001‐supinfo.docx.

## Data Availability

The data that support the findings of this study are available from National Cancer Database. Restrictions apply to the availability of these data, which were used under license for this study. Data are available from https://www.facs.org/quality‐programs/cancer‐programs/national‐cancer‐database/ with the permission of National Cancer Database.
