# Supplementary material for: Mitigating Disparities in Prostate Cancer Survival Prediction Through Fairness‐Aware Machine Learning Models
Source: Cancer Med. 2026 Jan 27;15(2):e71544. doi: 10.1002/cam4.71544 (PMC12835780; doi:10.1002/cam4.71544)

# Supplementary Material

Supplemental Figure 1: Selection of patients from National Cancer Database for development and evaluation of machine learning model to predict overall survival after radical prostatectomy.
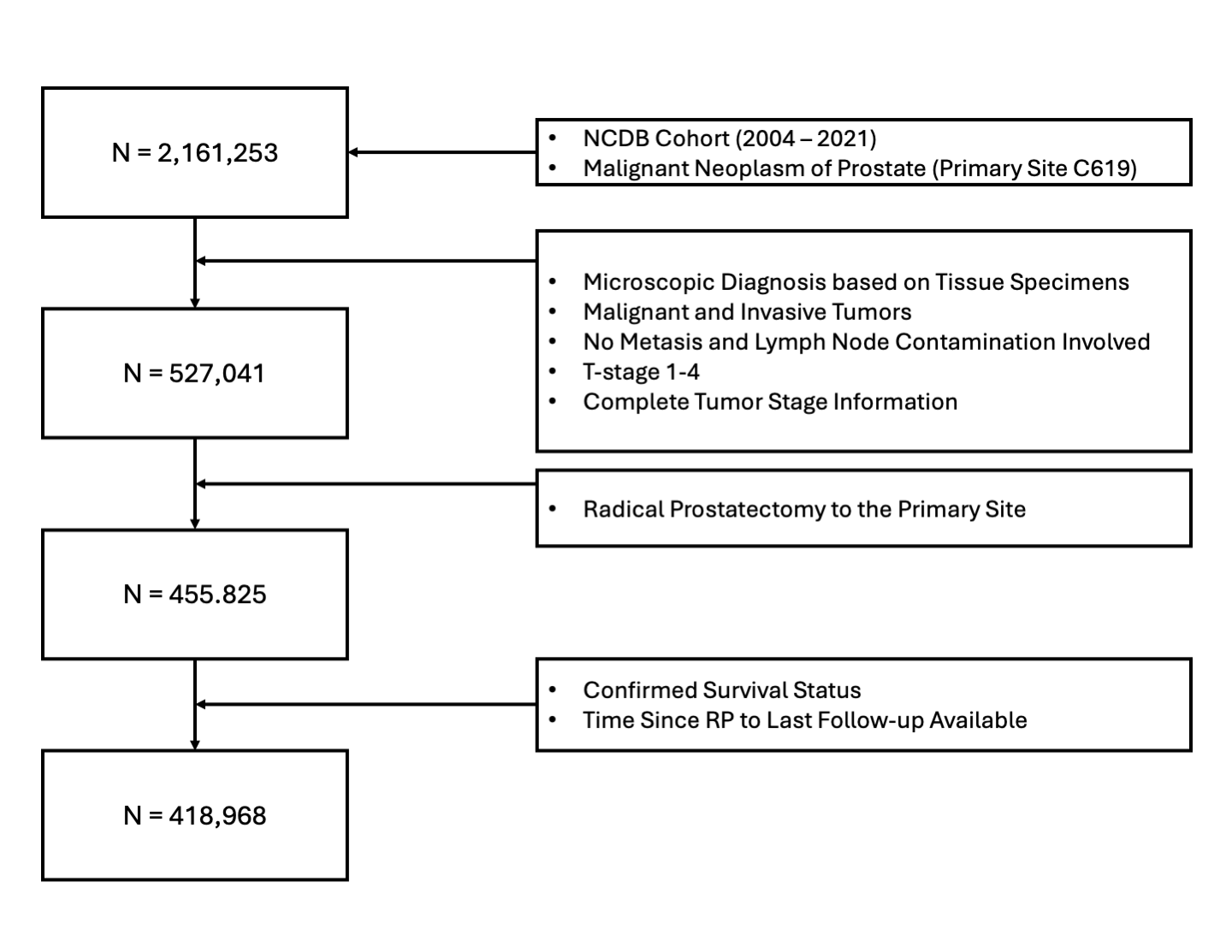

Supplement: Supplementary file 1 — Figure S1: cam471544‐sup‐0001‐supinfo.docx. [file CAM4-15-e71544-s001.docx]
